# Hydroxycitric Acid Inhibits Chronic Myelogenous Leukemia Growth through Activation of AMPK and mTOR Pathway

**DOI:** 10.3390/nu14132669

**Published:** 2022-06-27

**Authors:** Doriana Verrelli, Luca Dallera, Massimo Stendardo, Silvia Monzani, Sebastiano Pasqualato, Marco Giorgio, Rani Pallavi

**Affiliations:** 1 IRCCS European Institute of Oncology, Via Ripamonti 435, 20141 Milan, Italy; doriana.verrelli@gmail.com (D.V.); luca.dallera95@gmail.com (L.D.); massimo.stendardo@ieo.it (M.S.); silvia.monzani@ieo.it (S.M.); sebastiano.pasqualato@ieo.it (S.P.); 2 Department of Biomedical Sciences, University of Padova, Via Ugo Bassi 58/B, 35131 Padova, Italy

**Keywords:** nutraceuticals, hydroxycitric acid, AMPK, CML

## Abstract

Metabolic regulation of cancer cell growth via AMP-activated protein kinase (AMPK) activation is a widely studied strategy for cancer treatment, including leukemias. Recent notions that naturally occurring compounds might have AMPK activity led to the search for nutraceuticals with potential AMPK-stimulating activity. We found that hydroxycitric acid (HCA), a natural, safe bioactive from the plant *Garcinia gummi-gutta* (*cambogia*), has potent AMPK activity in chronic myelogenous leukemia (CML) cell line K562. HCA is a known competitive inhibitor of ATP citrate lyase (ACLY) and is widely used as a weight loss inducer. We found that HCA was able to inhibit the growth of K562 cells in in vitro and in vivo xenograft models. At the mechanistic level, we identified a direct interaction between AMPK and ACLY that seems to be sensitive to HCA treatment. Additionally, HCA treatment resulted in the co-activation of AMPK and the mammalian target of rapamycin (mTOR) pathways. Moreover, we found an enhanced unfolded protein response as observed by activation of the eIF2α/ATF4 pathway that could explain the induction of cell cycle arrest at the G2/M phase and DNA fragmentation upon HCA treatment in K562 cells. Overall, these findings suggest HCA as a nutraceutical approach for the treatment of CMLs.

## 1. Introduction

AMP-activated protein kinase (AMPK), once activated by energy stress, maintains cellular energy homeostasis by switching off almost all anabolic pathways, such as fatty acid, phospholipid, protein, and ribosomal RNA synthesis, that are required for cell growth. Conversely, AMPK has a promoting effect on catabolic processes such as fatty acid oxidation and mitochondrial biogenesis [1,2]. In general, AMPK activation restrains aerobic glycolysis used by most proliferative cells and promotes ATP generation by oxidative metabolism [3]. This effect of AMPK counteracts the so-called “Warburg” effect of cancer, a characteristic feature of many rapidly growing transformed cells that rely on glycolysis and lactate fermentation [4]. In addition, AMPK activation can dramatically affect many cellular processes relevant to carcinogenesis and cancer progression, such as mTOR signaling and inflammation [5]. Pertaining to this characteristic feature, many direct or indirect activators of AMPK, such as AICAR, metformin, and resveratrol, showed a promising effect in cancer treatment in both in vitro and in vivo cancer models [5]. Conversely, germline mutation in LKB1, the upstream activator of AMPK, has been associated with a higher prevalence of cancer [6]. Although AMPK could also have a tumor-promotive role, its role as a tumor suppressor has been an attractive therapeutic interest in cancer treatment and it has been found to oppose tumor progression in several cancer types [5,7,8]. AMPK can be activated by LKB1, CaMKK, and TGF-β in response to an increase in AMP/ATP ratio, low glucose, and energy stress [9,10]. Additionally, the administration of drugs and many naturally occurring compounds such as metformin, polyphenols, flavonoids, curcumin, and Chinese herbal compounds can activate AMPK [5,11,12]. The availability of diverse non-toxic nutraceuticals with AMPK activation properties provides an additional benefit in the field of cancer treatment as adjuvant therapy [13,14].

Chronic myeloid leukemia (CML) accounts for 15% of newly diagnosed cases of leukemia in adults [15]. Although tyrosine kinase inhibitors (TKIs) such as imatinib (IM) and its derivatives have significantly improved the clinical outcome of CML patients, many patients eventually develop resistance to TKIs [16]. Third-generation TKI, ponatinib, although often effective in resistant CMLs, has limited clinical utility due to its toxicity [17]. In this regard, there is an urgent need to develop alternative therapeutic strategies for CMLs. Interestingly, the AMPK pathway has been shown to have therapeutic value in CMLs [18,19]. The remarkable anti-leukemic effect of AMPK activators such as AICAR, metformin, and resveratrol, both in imatinib mesylate sensitive or resistant CMLs, indicates the therapeutic potential of AMPK activators in CMLs [20,21,22]. Hydroxycitric acid (HCA) is a natural extract from the Indian fruit Garcinia gummi-gutta, widely used for weight loss and blood cholesterol reduction in humans [23]. The 1,2-dihydroxilated form of citric acid behaves as a competitive inhibitor of the cytosolic enzyme adenosine triphosphate citrate lyase (ACLY) and thus interferes with the production of acetyl-CoA within the cytosol from citrate and oxaloacetate [24].

In this study, we characterized the effect of HCA on the AMPK/mTOR pathway and CML cell growth.

## 2. Materials and Methods

### 2.1. Cell Lines

K562, CML-T1, SKH-1, MEG-01, and KYO-1 chronic myelogenous leukemia cells were grown at 37 °C under 10% CO_2_ in RPMI 1640 medium (Gibco BRL, Paisley, UK) supplemented with 10% fetal bovine serum (20% FBS for KYO-1) (Euroclone, Pero, Milano, Italy), 2 mM L-glutamine, 100 U/mL penicillin, and 100 mg/mL streptomycin (Euroclone, Pero, Milano, Italy). Hydroxycitric acid tripotassium salt (HCA) (59847) was purchased from Sigma Aldrich (St. Louis, MO, USA).

### 2.2. Proliferation Assay

CellTiter-Glo^®^ luminescent cell viability assay (Promega Corporation, Madison, WI, USA) was performed according to the manufacturer’s guidelines. Briefly, cells were plated in triplicate at 2 × 10^5^ per well and treated with HCA (1–100 mM) for 72 h. Means and standard deviations generated from three independent experiments are reported as the percentage of viable cells. The IC50 value was calculated with GraphPad Prism software (version 8; GraphPad Software, San Diego, CA, USA).

### 2.3. Quantification of Apoptotic Cells

K562 cells were cultured in six-well plates at a cell density of 2 × 10^5^ cells per well with 1 mM and 5 mM of HCA for 72 h. The cells were collected, spun down at 240× *g*, and then analyzed by FACS for the presence of apoptotic cells using the annexin V-PE kit (Bender Medsystems, Wien, Austria), following the manufacturer’s instructions.

### 2.4. DNA Fragmentation

The K562 cells were collected and lysed in lysis buffer (200 μL) containing 10 mM Tris-Cl pH 7.5, 5 mM EDTA, and 0.2% Triton X-100. Lysates were treated for 60 min with 100 μg/mL RNase and then incubated for 60 min with 100 g/mL proteinase K at 37 °C. Cellular DNA was ethanol-precipitated, dried, and resuspended in Tris–EDTA buffer (10 mM Tris-Cl pH 8.0 and 1 mM EDTA). DNA was analyzed by electrophoresis on 1.4% agarose gels.

### 2.5. Cell Cycle Analysis

K562 cells were seeded in six-well plates (2 × 10^5^ cells each well), and treated with different concentrations of HCA for 48 and 72 h. Cells were collected, washed once in phosphate buffer solution (PBS; 137 mM NaCl, 2.7 mM KCl, 4.3 mM Na2HPO4, and 1.47 mM KH2PO4 pH 7.4) and 1% bovine serum albumin (BSA), and fixed by adding 70% cold ethanol in a dropwise manner. Overnight-fixed cells were treated with ribonuclease (RNase) (25 μg/mL) for 1 h at 37 °C, and stained with propidium iodide solution (100 μg/mL) for 30 min in the dark. The cell cycle distribution was detected with a flow cytometry system (BD FACS Celesta), and the data were analyzed with ModFit LT software (FlowJo v10, Ashland, OR, USA).

### 2.6. Protein Extraction and Western Blotting

Total protein extracts from cells were obtained by lysing K562 cells treated with 0.05 and 1M HCA in cold lysis buffer (50 mM Tris-HCl pH 8.0, 150 mM NaCl, 0.1% SDS, 1% NP-40, and 0.5% deoxycholic acid), in the presence of EDTA free protease inhibitors (Roche, Basel, Switzerland). Proteins were quantified by Bio-Rad protein assay (Bio-Rad Laboratories, Hercules, CA, USA). An equal amount of protein from each condition was separated on SDS-PAGE (12% gels) and then subsequently blotted onto PVDF membranes following conventional protocols. Finally, blots were blocked in 5% BSA, 1% Tween 20 in TBS (20 mM Tris-Cl and 150 mM NaCl, pH 7.5) at 4 °C overnight. The blots were probed with primary antibody specific to AMPKα (Cell Signaling, Danvers, MA, USA # 2535S; 1:1000), p-AMPKα (T177; Cell Signaling, Danvers, MA, USA # 2531S; 1:1000), S6 ribosomal protein (Cell Signaling, Danvers, MA, USA # 2217S; 1:1000), phospho-S6 ribosomal protein (Cell Signaling, Danvers, MA, USA; # 2211S; 1:1000), p-70 S6 kinase (Cell Signaling, Danvers, MA, USA # 2708S; 1:1000), phospho p-70 S6 kinase (Cell Signaling, Danvers, MA, USA # 9205S; 1:1000) ACLY (Cell Signaling, Danvers, MA, USA; # 4332S; 1:1000), EIF2α (Cell Signaling, Danvers, MA, USA; # 9722S; 1:1000), phospho-EIF2α (Cell Signaling, Danvers, MA, USA; # 3398S; 1:1000), ATF4 (Cell Signaling, Danvers, MA, USA; # 11815S; 1:1000), phospho-ACC (Cell Signaling, Danvers, MA, USA; # 11818S; 1:1000), LKB1 (Cell Signaling, Danvers, MA, USA; # 3047S; 1:1000); caspase 3 (Cell Signaling, Danvers, MA, USA; # 9662S; 1:1000), LC3 (Nanotools, Munich, Germany; # 0231-100BIOTIN/LC3-5F10; 1 μg/mL), and vinculin (Sigma-Aldrich, St. Louis, Missouri, United States; V9131: 1:5000) and secondary anti-rabbit IgG-HRP or anti-mouse IgG-HRP. Visualization of protein was performed by enhanced chemiluminescence (ECL) using the ChemiDoc Imaging system (BioRad, Hercules, CA, USA). In most of the experiments, each sample was loaded in duplicate, and probed by an antibody against either the phosphorylated form or total protein to avoid re-probing the same blot. Antibody against vinculin was used as a protein loading control. Densitometrical analysis of the Western blot images was performed using ImageJ 1.52. In brief, the band intensities were evaluated as the optical density and were then represented as fold change for HCA treated vs. untreated cells normalized for the loading control. Vinculin was used as the loading control.

### 2.7. Immunoprecipitation

HCA-treated and untreated K562 cells were lysed in immunoprecipitation lysis buffer (20 mM HEPES pH 7.4, 150 mM NaCl, 1 mM EDTA, 1 mM EGTA, 1% Triton-100, 0.5% sodium deoxycholate, 2 mM Na3VO4, 100 mM NaF) supplemented with protease inhibitor. Endogenous AMPK was immunoprecipitated using AMPKα antibody (Cell-Signaling, Danvers, MA, USA; #2523S). In brief, 2 mg of total protein was incubated with 5 L of AMPKα antibody at 4 °C for 4 h, followed by incubation with protein A-Sepharose beads for 3–4 h, at 4 °C. After washing them thoroughly, immune complexes were eluted from the beads by boiling them in Laemmili buffer and were separated on SDS-PAGE (12% gels) and analyzed by Western blot analysis with antibody against AMPK (Cell-Signaling, Danvers, MA, USA; #2523S; 1:1000) and ACLY (Cell-Signaling, Danvers, MA, USA, #4332S; 1:1000).

### 2.8. Size-Exclusion Chromatography

The K562 cell lysate treated with 1 mM HCA or untreated control was loaded onto a Superose 6 10/300 column (Cytiva, Marlborough, MA, USA) and eluted with a 0.4 mL/min flow of 50 mM Hepes pH 7.5, 150 mM NaCl, 2 mM EDTA, 1 mM DTT, in 400 μL fractions. Then, 40 μL from each fraction was separated on SDS-PAGE (12% gels) and analyzed by Western blot analysis with antibody against AMPK, LKB1, and ACLY (Cell-Signaling, Danvers, MA, USA; #4332S; 1:1000).

### 2.9. Animal Studies

All aspects of the animal experiment were performed in accordance with EU directives on the use of animals for experimental purposes and it was approved by the internal ethical committee and the Italian Ministry of Health (Project number 35/2016 and 130/17). NOD.Cg-PrkdcscidIl2rgtm1WjI/SzJ (NSG) were purchased from Charles River Laboratories Italia and maintained in our animal facility (European Institute of Oncology Cogentech Facility) under strict pathogen-free conditions, receiving sterilized pellets (VRF1 (P); Special Diet Services; #801900) and water ad libitum.

K562 cells (0.5 × 10^6^ in 100 μL of 1 × HBSS) with 15% of white Matrigel (Corning^®^ Matrigel^®^; CLS356231) were injected subcutaneously into the right flank of the 8–10-week-old male NSG mice. After 4 days of injection, mice were randomly divided into two groups, a control group (*n* = 7 mice) and a treatment group (*n* = 8 mice). The control group mice were given water by gavage and the treated group mice were given HCA dissolved in water at 3 mg/kg body weight by gavage daily throughout the experimental duration. Tumors were measured bidirectionally thrice weekly with calipers, and tumor volumes were calculated by the formula [*1*/2 (length × width^2^)], where length represents the largest tumor diameter and width represents the smallest tumor diameter. Mean tumor volumes were calculated from measurements performed on 7–8 mice in each group. After 25 days, the mice were sacrificed using an overdose of inhaled CO2, the tumor was removed, and tumor measurements and tumor weight were taken.

### 2.10. Statistical Analysis

The Student *t*-test was used to compare the mean differences between samples using Prism9. Statistical significance is indicated as follows: *p* > 0.05, *: *p* ≤ 0.05, **: *p* ≤ 0.01, ***: *p* ≤ 0.001, ****: *p* ≤ 0.0001.

## 3. Results

### 3.1. Hydroxycitric Acid Promotes AMPK Phosphorylation in CML Cells

HCA was selected along with other nutraceuticals with established or probable AMPK phosphorylation activity after screening in human liver cell lines. Five of these nutraceuticals (Table 1), including HCA, were tested directly in K562 cells to identify nontoxic treatments to combat emerging resistance in CMLs [18,19].

**Figure 1:**
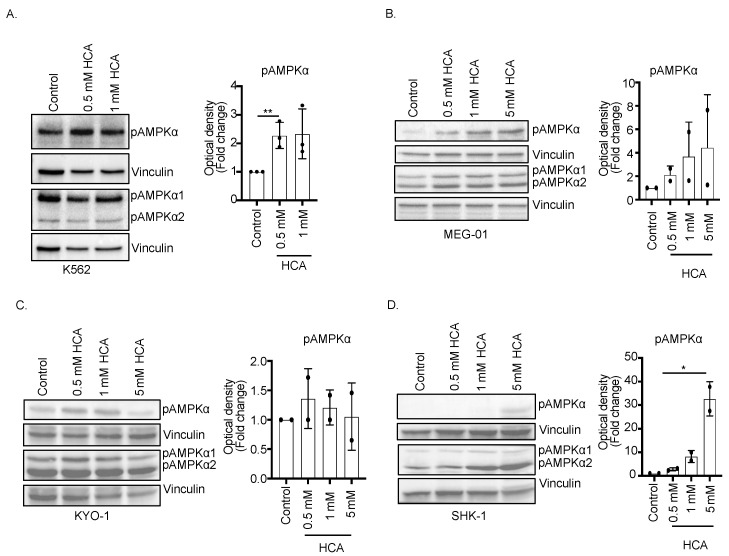
Hydroxycitric acid promotes AMPK phosphorylation in CML cells. CML cell lines were treated for 24 h with different concentrations of HCA. An equal amount of protein from each condition was separated on SDS PAGE (12% gel) and an immunoblot was performed using specific antibodies against pT172 AMPK and total AMPK. Vinculin was used as an internal control. (**A**) K562 (**B**) MEG-01 (**C**) KYO-1 (**D**) SHK-1. For the K562, MEG-01, KYO-1, and SHK-1, samples were loaded in duplicate. One was probed with total AMPK and the another one was probed with pT172 AMPK. Upper vinculin is referred to as pAMPK, and lower vinculin is referred to as the total AMPK. The bar graph beside each figure panel reflects the band intensity evaluated as optical density and represented as fold change for treated vs. untreated cells normalized for vinculin. ** *p* < 0.01, * *p* < 0.05 treated vs. untreated cells.

K562 cells were treated with different concentrations of natural extract for 24–48 h and the extent of AMPK T172 phosphorylation was revealed by Western blot. HCA, in particular, was found to be effective in stimulating AMPK phosphorylation without affecting the expression of AMPK (Figure 1A). Protocatechuic acid (PCA) and quercetin, although increasing the level of AMPK T172 phosphorylation, decreased the total AMPK level. Quercetin at higher concentrations even inhibited the phosphorylation of AMPK. Kuromanin chloride had a very moderate effect on AMPK T172 phosphorylation and total AMPK levels (Figure S1A–D).

Similarly, the HCA increased AMPK phosphorylation in the other four CML cell lines: MEG-01, KYO-1, and SKH-1 (Figure 1B–D). These results indicate that HCA is a relatively potent activator of AMPK in CMLs.

### 3.2. ACLY, a Direct Target of Hydroxycitric Acid, Interacts with AMPK

HCA is abundant in the peel of the Indian fruit Garcinia cambogia, whose extract is widely used for weight loss and blood cholesterol reduction in humans [23]. HCA behaves as a competitive inhibitor of adenosine triphosphate citrate lyase (ACLY), affecting the production of cytosolic acetyl-CoA that is required for fatty acid and cholesterol biosynthesis [24,25]. Further, ACLY physically interacts with AMPK and inhibits its phosphorylation at T172 residue [26]. Thus, we hypothesize that HCA, interfering with the ACLY activity, may affect the ACLY and AMPK association as well.

**Figure 2:**
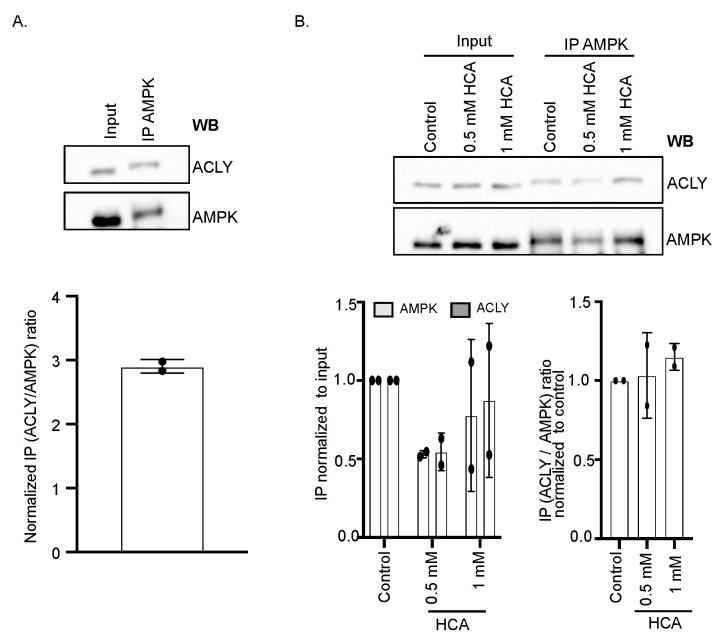
AMPK interacts with ACLY. (**A**) Co-immunoprecipitation of AMPK and ACLY in K562 cells. Endogenous AMPK was immunoprecipitated with antibody against total AMPK followed by Western blotting with anti-AMPK and anti-ACLY (upper panel). Bar graph showing the ratio of optical density of immunoprecipitated (IP) band of ACLY and AMPK after normalization with the respective input (lower panel). (**B**) Co-immunoprecipitation of AMPK and ACLY in K562 cells upon treatment with HCA (upper panel). Bar graph of optical density of immunoprecipitated ACLY and AMPK in each condition normalized to respective input band (left lower panel). Ratio of optical density of immunoprecipitated (IP) band of ACLY and AMPK after normalization with the control (right lower panel).

As expected, immunoprecipitation of endogenous AMPK1 by using AMPK antibody was able to pull down ACLY from the K562 cell lysate (Figure 2A). The treatment of K562 cells with 0.5 mM and 1 mM HCA seems to decrease the total level of pooled down AMPK–ACLY complex (Figure 2B, up). Under HCA treatment, the immunoprecipitated level of both AMPK and ACLY was less as compared to the untreated control. This was also reflected in the densitometry analysis of immunoprecipitated ACLY and AMPK protein bands normalized to the corresponding inputs at 0.5 μM HCA (Figure 2B, down). To further examine if there were any changes in the immunoprecipitated amount of ACLY to immunoprecipitated AMPK under HCA treatment, we evaluated the optical density of the immunoprecipitated proteins. We represented this as the fold change for HCA-treated vs. non-treated control cells normalized for the control (Figure 2B, down). There was no difference in the immunoprecipitated ACLY between the control and the treatment group, hence suggesting no effect of HCA on AMPK–ACLY interaction. To provide further evidence that AMPK and ACLY are part of a complex, we examined their co-elution using size-exclusion chromatography. We found a fraction of AMPK to co-elute with ACLY, suggesting that AMPK and ACLY are part of a similar migrating protein complex (Figure S2). Here, too, HCA treatment had no major effect on the migration of protein complexes. Although we found a direct interaction between AMPK and ACLY, the direct target of HCA, HCA seems not to affect the interaction between AMPK and ACLY. Direct experiments using purified full-length ACLY and AMPK with or without HCA will be required to characterize this interaction in detail biochemically.

### 3.3. Concurrent Activation of AMPK and mTOR Pathway Increases Metabolic Stress Pathway

**Figure 3:**
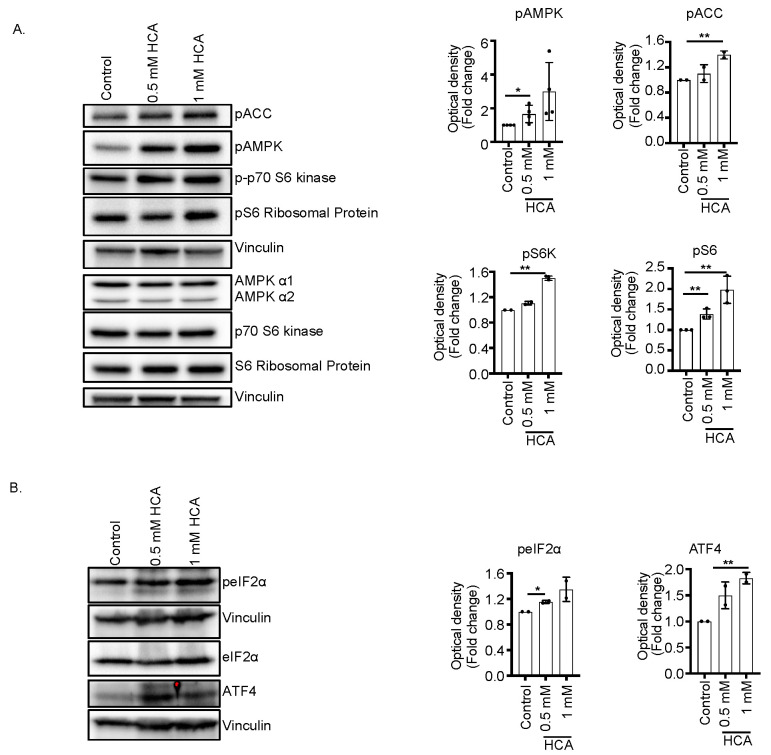
HCA stimulates unfolded protein response pathway. Cell lysate was prepared from K562 cells treated with indicated concentration of HCA. Proteins were separated and immunoblotted with antibody against desired protein. (**A**) HCA induces concomitant activation of AMPK and mTORC1 pathways in K562 cells. Samples were loaded in duplicate. One of them was used to immunoblot with phosphor form and the other was used to immunoblot respective total protein. Upper vinculin is referred to as p-AMPK, p-p70S6K, and pS6 ribosomal protein; lower vinculin is referred to as total AMPK, p70S6K, and S6 ribosomal protein. (**B**) HCA-treated K562 show upregulation of unfolded protein response markers ATF4 and p-elF2α. Upper vinculin is referred to as peIF2α and ATF4; lower vinculin is referred to as eIF2α. The bar graph beside each figure panel reflects the band intensity evaluated as optical density and represented as fold change for treated vs. untreated cells normalized for vinculin. ** *p* < 0.01, * *p* < 0.05 treated vs. untreated cells.

Since AMPK activation has been associated with the inhibition of mTOR, we analyzed the state of the mTOR pathway upon HCA treatment. Results revealed that treatment with 0.5 or 1 mM HCA increases the phosphorylation level of ribosomal protein S6 Kinase (S6K), a known downstream effector of mTOR complex 1. In addition, the level of phosphorylation of S6, a direct substrate of the S6K, was significantly increased in HCA-treated samples, confirming the consistent activation of the mTOR pathway by HCA (Figure 3A). This indicated that HCA treatment activated both AMPK and mTORC1/S6K pathways. The concurrent activation of AMPK and mTORC1 was also described upon amino acid load [27], and in myeloid leukemia following treatment with the AMPK inducer (GSK621), which seems to enhance the unfolded protein response (UPR) [28]. Therefore, we examined the phosphorylation of eIF2α, a marker for UPR activation and ATF4, the terminal effector of the eIF2α pathway (Figure 3B), in K562 cells treated with HCA. The results indicated that HCA treatment increased the level of phosphorylated eIF2α and ATF4, indicating that treatment with HCA triggered UPR.

### 3.4. HCA Induces Cell Cycle Arrest at the G2/M Phase in K562 Cells

**Figure 4:**
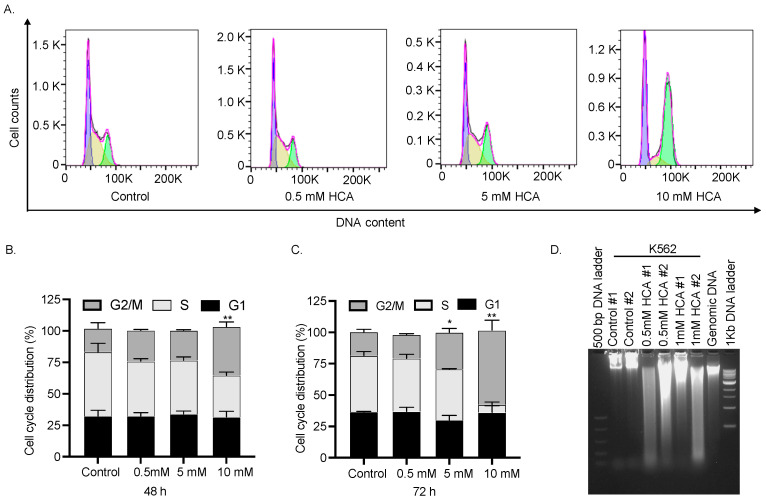
HCA induces DNA fragmentation and G2/M arrest in K562 cells. (**A**) K562 cells were treated for 48 h and 72 h with different concentrations of HCA, as indicated. Cells were collected, treated with RNase, and stained with PI. Cell cycle distribution was then detected through flow cytometry system (BD FACS Celesta). Graphs were obtained with data analysis through ModFit LT software (FlowJo v10, Ashland, OR, USA). The fitted cell populations in G1, S and G2/M phases are represented in purple, light yellow, and light green. (**B**) Bar graph showing cell cycle distribution at 48 h. Treatment with 10 mM HCA leads to a significant delay in the cell cycle progression at the G2/M phase. ** *p* < 0.01; unpaired *t*-test (**C**) Bar graph showing cell cycle distribution at 72 h. Treatment with 5 mM and 10 mM HCA lead to a significant delay in the cell cycle progression at G2/M phase. (* *p* < 0.05; ** *p* < 0.01; unpaired *t*-test (**D**) K562 cells were treated with different concentrations of HCA, as indicated. Samples were lysed and prepared following the appropriate protocol and then DNA fragmentation was evaluated by agarose gel electrophoresis (2%). M, 500 bp DNA ladder; DNA, genomic DNA standard; L, 1 kb DNA ladder.

The UPR activation boosts autophagy and/or triggers apoptosis [29]. However, treatment with 0.5–5 μM HCA for 24 h or 48 h had no effect on the autophagic marker protein LC3 and did not show any induction of apoptosis as analyzed by the level of cleaved caspase-3 or by the quantification of annexin V/PI staining by flow cytometry in K562 cells (Figure S3A–E). However, 5–10 mM HCA treatment starting from 48 h resulted in an accumulation of K562 cells at the G2/M phase of the cell cycle (Figure 4A). Notably, HCA-treated cells showed DNA fragmentation, which might account for the G2/M blockage (Figure 4B). However, we were not able to decipher the type of cell death produced by the HCA treatment. Our data suggest that the major consequences of the treatment with HCA on K562 cells are G2/M blockage and DNA fragmentation. Further, it seems that HCA leads to a caspase-independent DNA fragmentation, as described in the ischemic response [30], and is different from other anti-cancer nutraceuticals, such as quercetin or Ruta graveolens, that are reported to induce DNA fragmentation through a typical caspase-dependent mechanism [31,32]. However, the involvement of necroptosis or another caspase-independent DNA fragmentation-associated cell death could not be ruled out and requires further investigation.

### 3.5. HCA Inhibited Tumor Cell Growth In Vitro and In Vivo

**Figure 5:**
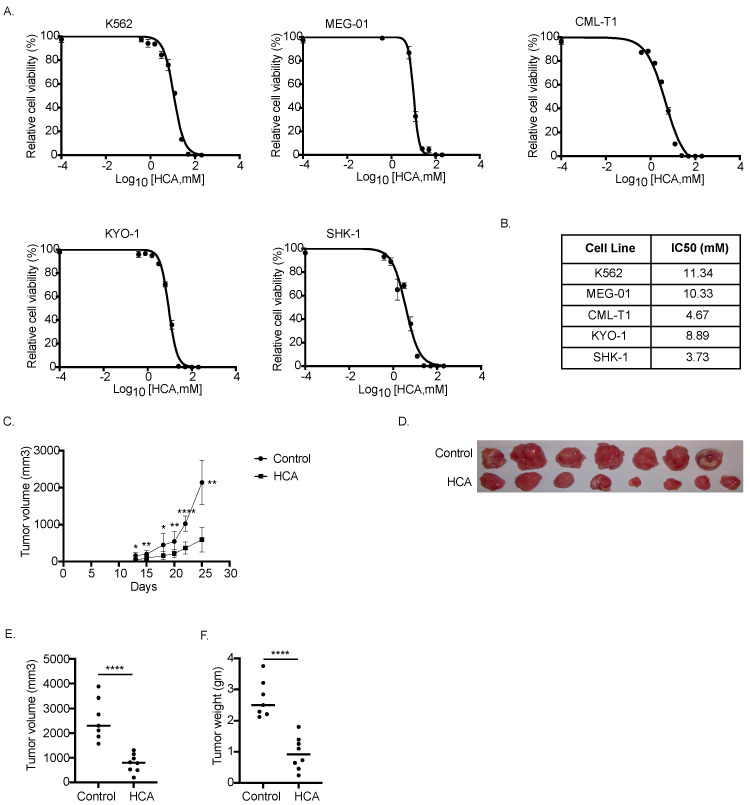
HCA inhibits K562 cells’ growth in vitro and in vivo. (**A**) CML cell lines (K562, MEG-01, CML-T1, KYO-1, and SHK-1) were incubated with different concentrations of HCA. After 72 h, cell viability was measured and response curves were plotted using GraphPad Prism 9. (**B**) List of IC_50_ values in mM calculated from curve ‘A’. (**C**–**E**) NSG mice were subcutaneously injected at the right flank with 0.5 × 10^6^ K562 cells and were divided randomly into control and HCA-treated group. HCA was administered daily by oral gavage at 3 mg/kg body weight. (**C**) Plot showing tumor volume with time. HCA-treated mice showed a significant delay in the tumor growth (* *p* < 0.05; ** *p* < 0.01; **** *p* < 0.0001; unpaired *t*-test). (**D**) Image of tumor from control and HCA-treated mice at the time of sacrifice. (**E**) Comparison of tumor volume of control (*n* = 7) and HCA-treated mice (*n* = 8) (*p* < 0.0001; unpaired *t*-test). (**F**) Comparison of tumor weight of control (*n* = 7) and HCA-treated mice (*n* = 8) (*p* < 0.0001; unpaired *t*-test).

To validate the therapeutic potential of HCA on CML, we evaluated first the effect of HCA on CML cells in vitro growth. Briefly, K562 cells were incubated with different concentrations of HCA ranging from 1 mM to 100 mM of HCA up to 72 h, and the number of viable cells in the culture was quantified by CellTiter-Glo. Results indicated that HCA inhibited K562 cell proliferation in a concentration-dependent manner, with an IC50 of 11.34 mM (Figure 5A). Consistently, HCA retarded the growth of several other human CML cell lines, including MEG-01, CML-T1, SKH-1, and KYO-1, with an IC50 in the 3–12 mM range (SKH-1, 3.73 mM; CML-T1, 4.67 mM; KYO-1, 8.89; MEG-01, 10.33) (Figure 5A). Nevertheless, HCA did not affect cellular proliferation in normal mouse embryo fibroblasts, even at a concentration as high as 100 mM.

To validate in vivo the antileukemic effect of HCA, we investigated the effect of supplementation by gavage of 3 mg/kg body weight of HCA on a xenograft mouse model generated by injecting K562 cells subcutaneously in NSG mice. After 4 days of transplantation, mice were treated with HCA (3 mg/kg body weight in water) or water by gavage once daily throughout the experimental duration. K562 growth in vivo was followed for 25 days and tumor size was measured thrice a week. Interestingly, the HCA group showed reduced tumor growth in comparison to vehicle-treated mice (Figure 5B). Moreover, the excised tumor from the HCA-treated mice looked visually smaller as compared to non-treated mice (Figure 5C). When compared with the final average tumor volume of control mice (2558 ± 843 mm^3^), there was a three-fold reduction in the average tumor volume (782 ± 367 mm^3^; *p* < 0.001) (Figure 5D) and a significant reduction in the tumor weight in HCA-treated mice (2.7 ± 0.6 g in control vs. 0.9 ± 0.5 g in HCA-treated; *p* < 0.0001) (Figure 5E). These findings demonstrate that HCA treatment inhibited CML growth in both in vitro and in vivo CML models.

## 4. Discussion

Nutraceuticals, natural plant products, are emerging as a key agent to activate the AMPK pathway, and many show potent antitumor activity [33]. Due to their very low or non-existent toxic effects, they represent a promising therapeutic alternative in cancer. Here, we examined the AMPK activity of the members of flavonoids and the widely used anti-obesity drug HCA in K562 cells. We found that HCA, a competitive inhibitor of ACLY, was able to promote AMPK phosphorylation at the T172 in K562 cells. HCA did not alter the total AMPK protein level, suggesting that the increased AMPK T172 phosphorylation observed upon HCA treatment was not due to the altered expression of AMPK. Activation of AMPK by HCA, without alteration in gene expression, was also observed in broiler chickens [34,35].

Interestingly, several studies suggested a possible relationship between ACLY inhibition and AMPK activation. An inverse effect on ACLY and AMPK activity was identified upon treatment with a structurally different compound, ETC-1002 (8-hydroxy-2, 2, 14, 14-tetramethylpentadecanedioic acid), that has been developed for the treatment of dyslipidemia. Treatment with ETC-1002, while it resulted in ACLY inhibition, promoted AMPK activity [36]. Interestingly, Migita et al. found that ACLY knockdown activates AMPK in cancer cells, and AMPK activation predicts the therapeutic response to ACLY knockdown in cancer cells [37]. The inhibitory effect of ACLY on AMPK activation was also described in primary human dermal fibroblasts, where the direct interaction of ACLY with AMPK was involved in suppressing the AMPK phosphorylation [26]. Similarly, we found AMPK to co-immunoprecipitate ACLY, indicating an interaction between AMPK and ACLY in K562 cells.

Generally, the anti-proliferative activity of AMPK has been attributed to the inhibition of the mTOR pathway. In fact, the first pharmacological activator of AMPK, 5-aminoimadazole-4-carboxamide ribonucleoside (AICAR), showed AMPK-dependent inhibition of mTOR signaling. Consistently, most naturally occurring or pharmacological AMPK activators such as metformin, resveratrol, honikiol, demethoxycurcumin, tanshinone IIA, and antroquinonol showed inhibition of the mTOR pathway [20,38,39,40,41,42]. However, we found that treatment with HCA leads to the paradoxical activation of the AMPK and mTOR pathways, suggesting a disconnection between the AMPK and mTOR axes. We found increased phosphorylation of S6K, S6 ribosome protein (markers for mTORC1 pathway activation), in addition to AMPK phosphorylation in HCA-treated K562 cells. Such disconnection within AMPK and its antagonist mTOR was also observed under amino acid (aa) sufficiency. Using computational modeling to identify other aa inputs to the mTOR network predicted independent aa input to the network via AMPK. The authors then experimentally showed that aa acutely activates AMPK concurrently with mTOR to possibly maintain protein homeostasis and deliver metabolite intermediates for the biosynthetic process [27]. Another study using an AMPK activator, thienopyridone-derived compound GSK621, in AML also supported the disconnection between AMPK and mTOR with sustained mTOR activation, even after AMPK activation by GSK621 [28].

Sustained mTOR activity has been shown to trigger an unfolded protein response [28,43]. As mTOR activity is critically linked to protein synthesis and the endoplasmic reticulum is a principal site for the folding and maturation of proteins, sustained mTOR activity may lead to the accumulation of unfolded protein and thus perturb ER homeostasis. In general, UPR mediators alleviate ER stress by attenuating protein synthesis, increasing the folding capacity of ER, and/or by degradation of the unfolded protein. However, unresolved ER stress inhibits cell cycle progression and kills the cells by inducing apoptosis, autophagy, necroptosis, or immunogenic cell death [44,45]. In our system, co-activation of the AMPK and mTOR pathway by HCA resulted in an ER stress response-mediated accumulation of cells in the G2/M phase of the cell cycle and DNA fragmentation. Although we did not find any evidence for caspase activation, DNA fragmentation was evident in HCA-treated samples. It could be possible that treatment with HCA leads to caspase-independent DNA fragmentation. In fact, in Leishmania, ER stress-induced apoptosis occurs through a caspase-independent mechanism [46]. Caspase-independent cell death is a recognizable phenomenon; however, our understanding of how cells actually die during this is limited due to the unavailability of proper means to detect caspase-independent death [47]. Possible involvement of necroptosis or another caspase-independent DNA fragmentation-associated cell death requires further investigation [48,49]. Finally, our finding that HCA treatment inhibits the growth of CML cell lines in vitro and a K562 xenograft in vivo suggests the therapeutic potential of HCA in CMLs.

## 5. Conclusions

Overall, our study suggests that co-activation of mTOR and AMPK could be a strategy to induce ER stress to combat cancer cells. In fact, the tumor-suppressive property of ER stress-induced UPR has been acknowledged, and many ER stress inducers are being tested in several cancer models [45]. Interestingly, many nutraceuticals have been shown to induce ER stress in cancer cells [50]. In addition, our study revealed a new therapeutic opportunity for the development of drugs targeting AMPK–ACLY. Targeting the AMPK–ACLY interaction could be a new therapeutic strategy for cancer treatment.

## Figures and Tables

**Table 1:** Nutraceuticals used in this study.

Nutraceuticals	Category	Food Source	Health Benefits
Cyanidine-3-O-glucoside chloride (Kuromanin chloride)	Flavonoids (Anthocyanin)	Berries such as blackberry, gooseberry, red raspberry, etc. Vegetables such as black olive, red lettuce, black beans	Cancer, inflammation, oxidative stress, and cardiovascular diseases
Hydroxycitric acid (HCA)	Organic acids	Fruit rinds of Garcinia; calyxes of hibiscus (used as a herbal tea	Weight loss, cancer
Protocatechuic acid (PCA)	Polyphenols	Olives, white wine grapes, calamondin citrus fruit	Cancer, inflammation, oxidative stress, and cardiovascular diseases
Quercetin	Flavonols	Red onions, kale, broccoli, berries, cherries, grapes, and citrus fruit	Diabetes, cancer, inflammation, aging, etc.
Naringenin	Citrus flavanones	Citrus fruits such as blood oranges, sour oranges, grapefruits, limes, mandarins, etc.	Cancer and cardiovascular diseases

## Data Availability

Not applicable.

## Supplementary Materials

The following supporting information can be downloaded at: https://www.mdpi.com/article/10.3390/nu14132669/s1, Figure S1: AMPK-phosphorylating activity of nutraceuticals; Figure S2: SEC elution profile of AMPK, ACLY, and LKB1; Figure S3: HCA induces neither apoptosis nor autophagy in K562 cells.
