# Supplementary material for: Hydroxycitric Acid Inhibits Chronic Myelogenous Leukemia Growth through Activation of AMPK and mTOR Pathway
Source: Nutrients. 2022 Jun 27;14(13):2669. doi: 10.3390/nu14132669 (PMC9268148; doi:10.3390/nu14132669)
Supplement: Supplementary file 1 [file nutrients-14-02669-s001.zip › nutrients-1747929-supplementary.pdf]

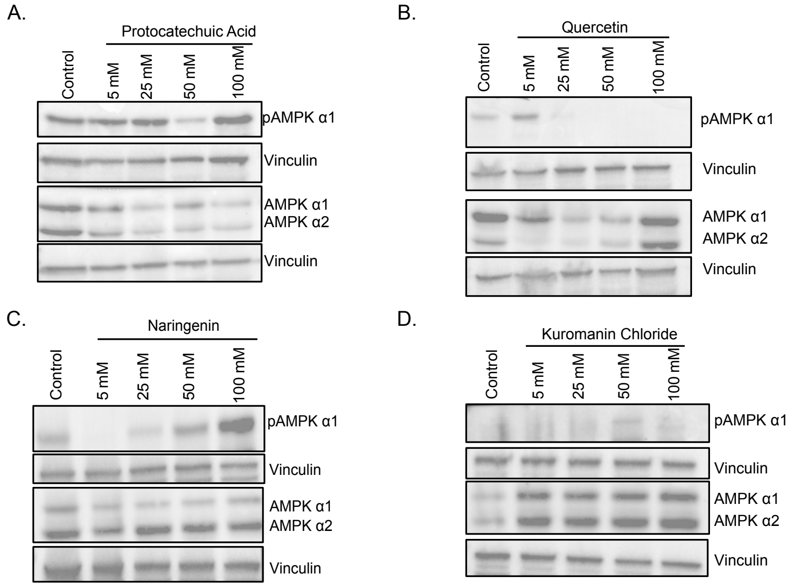


**Supplementary Figure S1:** AMPK- phosphorylating activity of nutraceuticals - K562 cells were treated for 24 h with different concentrations of the indicated compounds. Equal amount of protein from each condition was separated on SDS PAGE (12% gel) and protein expression levels were investigated through western blot analysis using antibody against phosphorylated and total AMPK. Vinculin has been used as an internal control. Upper vinculin is referred to pAMPK; lower vinculin is referred to total AMPK.

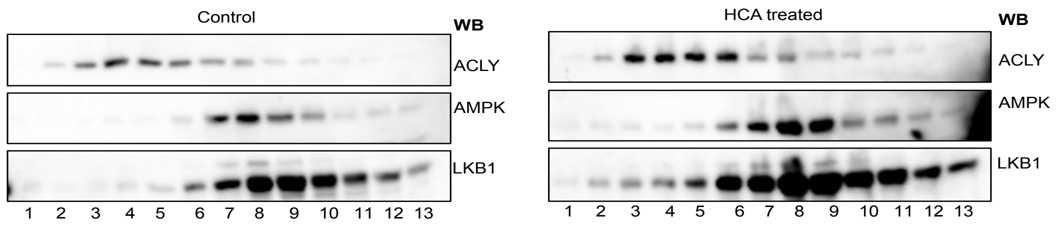


**Supplementary Figure S2:** SEC elution profile of the AMPK, ACLY and LKB1**:** SEC elution profile of the AMPK, ACLY and LKB1 in K562 cells under control (left panel) and HCA treatment (right panel). Protein lysate from HCA treated and control K562 cells was analysed by size-exclusion chromatography to examine if they are part of the same complex. ACLY peaks at the fractions 4 and 5 (corresponding to C7 and C8 of the Superose 6 column) and co-eluted with AMPK, which peaks at the fraction 8 (C11 of the Superose 6 column) (Figure 2C, left panel). This suggests that AMPK and ACLY are a part of complex. Although HCA treated samples ACLY showed broader peak ranging from fraction 3 to 6 (C6 to C9 of the Superose 6 column) and still it seems to be co-eluted with AMPK which peaks at fraction 8 and 9 (C12-D12 of the Superose 6 column) (Figure 2C, right panel). HCA treatment had no major effect on the elution profile of LKB1 (AMPK upstream kinase).

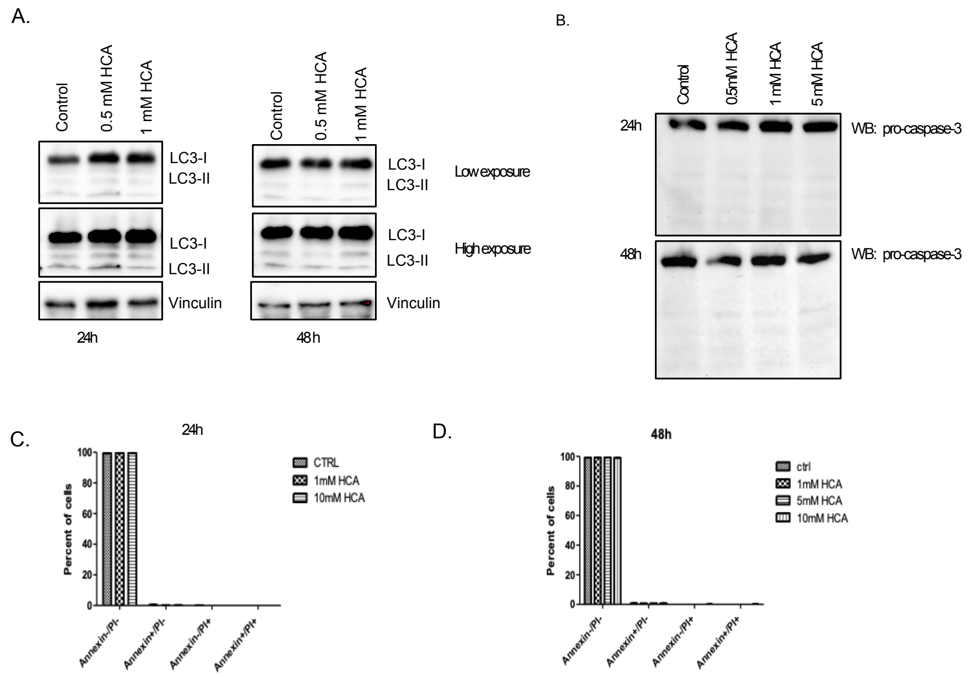


**Supplementary Figure S3.** HCA induces neither apoptosis nor autophagy in K562 cells. K562 cells were treated with different concentrations of HCA for 24 and 48 h, as indicated. (A) activation of autophagy was analyzed by immunoblotting for LC3. (B) activation of apoptosis was analyzed by immunoblotting for cleaved caspase 3. (C). The annexin V/PI staining was performed and analyzed by FACS.
